# Dissecting Zika virus transmission by *Aedes aegypti* and *Culex quinquefasciatus* mosquitoes to a vertebrate host and its coinfection with Mayaro virus

**DOI:** 10.3389/fmicb.2025.1724153

**Published:** 2026-01-28

**Authors:** Larissa Krokovsky, Carlos Ralph Batista Lins, Duschinka Ribeiro Duarte Guedes, Gabriel da Luz Wallau, Constância Flávia Junqueira Ayres, Marcelo Henrique Santos Paiva

**Affiliations:** 1 Departamento de Entomologia, Instituto Aggeu Magalhães (IAM/FIOCRUZ-PE), Caruaru, Brazil; 2 Biotério de Criação, Instituto Aggeu Magalhães (IAM/FIOCRUZ-PE), Recife, Brazil; 3 Núcleo de Bioinformática, Instituto Aggeu Magalhães (IAM/FIOCRUZ-PE), Recife, Brazil; 4 Universidade Federal de Santa Maria (UFSM), Santa Maria, Brazil; 5 Department of Arbovirology and Entomology, Bernhard Nocht Institute for Tropical Medicine, WHO Collaborating Center for Arbovirus and Hemorrhagic Fever Reference and Research, National Reference Center for Tropical Infectious Diseases, Hamburg, Germany; 6 Centro Acadêmico do Agreste (UFPE/CAA), Universidade Federal de Pernambuco, Recife, Brazil

**Keywords:** arbovirus, coinfection, mice, mosquitoes, vector competence

## Abstract

In recent years, the co-circulation of arboviruses including Zika (ZIKV), dengue (DENV), and chikungunya (CHIKV) has been increasingly reported worldwide, contributing to a complex and poorly understood epidemiological scenario. The simultaneous transmission of multiple arboviruses through a single mosquito bite is a documented phenomenon that can influence disease pathogenesis and modify transmission dynamics. This study investigated the dynamics of ZIKV infection in *Aedes aegypti* (RecLab) and *Culex quinquefasciatus* (CqSLab), and assessed the potential for ZIKV and Mayaro virus (MAYV) co-infection and co-transmission by *Ae. aegypti*. Mosquitoes were artificially blood-fed with ZIKV, MAYV, or both, and allowed to feed on immunodeficient mice. Clinical signs were monitored in mice, and infection was assessed using RT-qPCR. Finally, to complete the transmission cycle evaluation study, naive mosquitoes were subsequently fed on infected mice. Among 12 mice exposed to ZIKV-infected *Ae. aegypti*, all developed symptoms by days 7–8 post-exposure. Notably, mice bitten by co-infected *Ae. aegypti* displayed symptoms as early as days 2–3 post-exposure. Mosquitoes feeding on these mice showed high ZIKV infection rates, which were further elevated in the context of co-infection. Regarding the 12 mice exposed to *Cx. quinquefasciatus*, they showed no signs of infection, despite RT-qPCR positivity. These results validate a standardized murine model for arbovirus studies and reveal enhanced ZIKV transmission potential during co-infection with MAYV. The findings offer key insights into arbovirus co-infection dynamics and their potential impact on epidemiology and pathogenesis.

## Introduction

Mosquito-borne diseases continue to pose a major global public health challenge, which affects millions of individuals annually, and represents a burden further exacerbated by the ongoing spread and re-emergence of arboviruses in regions previously considered non-endemic (WHO, 2017). These pathogens are maintained in nature through cycles between hematophagous insects and vertebrate hosts (Weaver and Reisen, 2010). *Aedes aegypti* is considered the main vector of arboviruses, such as dengue (DENV), Zika (ZIKV), and chikungunya (CHIKV) viruses (Gubler, 2011; Vega-Rúa et al., 2014; Chouin-Carneiro et al., 2016). In an urban setting, these three viruses share human hosts and mosquito vectors, and their transmission is influenced by a number of factors, such as biological, economic and ecological, resulting in a strong epidemiological interaction (Gardner et al., 2018; Vogels et al., 2019). In addition, *Ae. aegypti* is also considered a competent vector for several zoonotic viruses, such as Yellow Fever (YFV) and Mayaro (MAYV) which predominantly circulate in the sylvatic environment (Souza-Neto et al., 2019). From 2015 to 2016, Brazil experienced a major epidemic of ZIKV which resulted in the characterization of a unique clinical profile in newborns (Congenital Zika Syndrome) and adults (Guillain Barret Syndrome) associated to this virus infection. Like *Ae. aegypti*, *Culex quinquefasciatus* mosquitoes play an important role as vectors of urban pathogens (Sudeep, 2014). In Brazil, this species is recognized as the main *Wuchereria bancrofti* vector and, has been implicated in the ZIKV transmission in both laboratory experiments and field surveillance (Guedes et al., 2017; Ministério da Saúde, 2021). In this particular scenario, *Cx. quinquefasctiatus* exhibits a marked anthropophilic feeding behavior and, in certain regions of the country, displays significantly higher intradomicile population densities compared to *Ae. aegypti* (Fontes et al., 2012).

The scenario of intense circulation of different arboviruses along with the abundant presence of vectors increases the probability of simultaneous infections in mosquitoes and humans, either simultaneously (coinfection) or in sequence (superinfection) and co-transmission (mosquito bite transmitting several viruses simultaneously) (Ciota, 2019; Vogels et al., 2019). Arbovirus coinfections and superinfections are increasingly recognized as relevant events in endemic regions where multiple viruses co-circulate and share competent mosquito vectors. These interactions can modify viral fitness, replication dynamics, and transmission potential in both mosquitoes and vertebrate hosts, ultimately influencing epidemic outcomes. Reports of simultaneous detection of ZIKV, MAYV, DENV, and CHIKV in mosquitoes and humans highlight the likelihood of mixed infections in nature and suggest that co-circulation may enhance or suppress specific viruses depending on the biological context (Carrillo-Hernández et al., 2018). Moreover, co-infected vectors may deliver multiple pathogens within a single bite, potentially altering host pathogenesis and immune responses, while superinfection scenarios reflect sequential exposures that more closely mimic natural transmission cycles. Therefore, understanding these interaction patterns is essential to improve risk assessment and surveillance strategies in areas experiencing overlapping arboviral outbreaks. Despite growing evidence, the mechanisms underlying virus–vector–host interactions are still poorly understood. Nevertheless, four theoretical scenarios of viral interaction have been proposed for both mosquitoes and humans: (i) enhanced replication of both viruses; (ii) reduced replication of both viruses; (iii) competitive suppression between co-infecting viruses; and (iv) absence of interaction (Rückert and Ebel, 2018; Vogels et al., 2019). Previous studies of vector competence have already demonstrated that the *Ae. aegypti* mosquito is capable of transmitting DENV, ZIKV, and CHIKV in double or triple infection during a single blood meal, under laboratory conditions (Rückert et al., 2017; Teixeira et al., 2021; Garcia et al., 2022). In addition to this combination of viruses, coinfection of MAYV and ZIKV by this species has been described using vertebrate cells and laboratory mosquitoes (Brustolin et al., 2023). Although rare, co-infection events in humans have already been reported in multiple countries with DENV, ZIKV and CHIKV (double or triple infection) (Acevedo et al., 2017; Carrillo-Hernández et al., 2018; Suwanmanee et al., 2018). In Brazil, neurological disorders were associated with a combination of coinfections, such as ZIKV and CHIKV (Brito et al., 2017), DENV and CHIVK (do Rosário et al., 2018), and DENV and MAYV (Zuchi et al., 2014).

Studies involving the transmission cycle of these viruses using mosquitoes and murine animal models (mice susceptible to infection) can mimic the vector infection in natural systems, but they are usually performed in single infection assays (Uraki et al., 2018; Baldon et al., 2022; Krokovsky et al., 2023). Arboviruses are strongly controlled by the type I interferon (IFN-I) system in mice, which limits viral replication and viremia to levels that are often undetectable in immunocompetent strains (Morrison and Diamond, 2017; Rosa et al., 2023). For this reason, several well-established mice models lacking IFN signaling, like C57 IFNAR BL/6, A129 and AG129 mice have become essential for experimental arbovirus research (Santiago et al., 2015; Lazear et al., 2016; Rossi et al., 2016). These models provide a permissive environment that allows systemic infection, detectable viremia, and the presentation of relevant clinical signs, enabling studies of virus replication, pathogenesis, and vertebrate-to-mosquito transmission. Given the recent outbreak involving multiple arboviruses, it is essential to investigate the transmission dynamics of these viruses in local vector species under co-infection scenarios, particularly in the context of ongoing viral introductions (Esposito and Fonseca, 2017; Diagne et al., 2020). Thus, the primary goal of the present study was to investigate the vector-host interaction (mosquito-mice-mosquito) between ZIKV, *Ae. aegypti*, *Cx. quinquefasciatus* and a vertebrate host, and the role of each species in the cycle. In addition, we have provided insights into the co-infection and co-transmission of ZIKV and MAYV viruses in *Ae. aegypti.*

## Materials and methods

### Viral strains and cell line

ZIKV (ZIKV/H.sapiens/Brazil/PE243/2015 - KX197192) and MAYV (MAYV/BR/Sinop/H307/2015 - MH513597) strains derived from human serum isolates were kindly provided by Dr. Marli Tenório (FIOCRUZ-PE) and Dr. Roberta Bronzoni (UFMT), respectively. Viral stocks were prepared by sample inoculation on an 80–90% confluent monolayer of VERO CCL81 cells. Monolayer was grown in a T-25 (25cm^2^) cell flasks containing Minimum Essential medium (MEM; Gibco, Grand Island, NY, USA) supplemented with 10% fetal bovine serum (FBS; Gibco, Grand Island, NY, USA), and 1% penicillin/streptomycin (Gibco, Grand Island, NY USA) in an incubator at 37 °C + 5% CO_2_. Up to 5 days after the inoculation of viruses, when cytopathic effects were visualized, cell cultures were centrifuged at 2,000 g for 10 min, supernatants were transferred to cryotubes, and then stored at −80 °C until use. Before mosquito infection, viral titer was calculated via plaque assay and reached 2 × 10^6^ plaque-forming units per milliliter (PFU/mL) for ZIKV (P6) and 1 × 10^8^ PFU/mL for MAYV (P4). Transmission assays were performed with a multiplicity of infection (MOI) of 0.1 for both viral stocks.

### Mosquito oral infection with ZIKV

Artificial feeding of mosquitoes was performed to initiate the first transmission cycle in mice. Both mosquito colonies used in the present study were obtained from natural populations collected in Recife, Brazil: the RecLab (*Ae. aegypti*) and the CqSLab (*Cx. quinquefasciatus*). These mosquitoes have been maintained at standard conditions of 26 ± 2 °C, 65–85% relative humidity, 10/14 light/dark cycle in the Entomology Department at the Aggeu Magalhães Institute (IAM) for several generations (de Araujo et al., 2007; Amorim et al., 2013).

**Figure 1:**
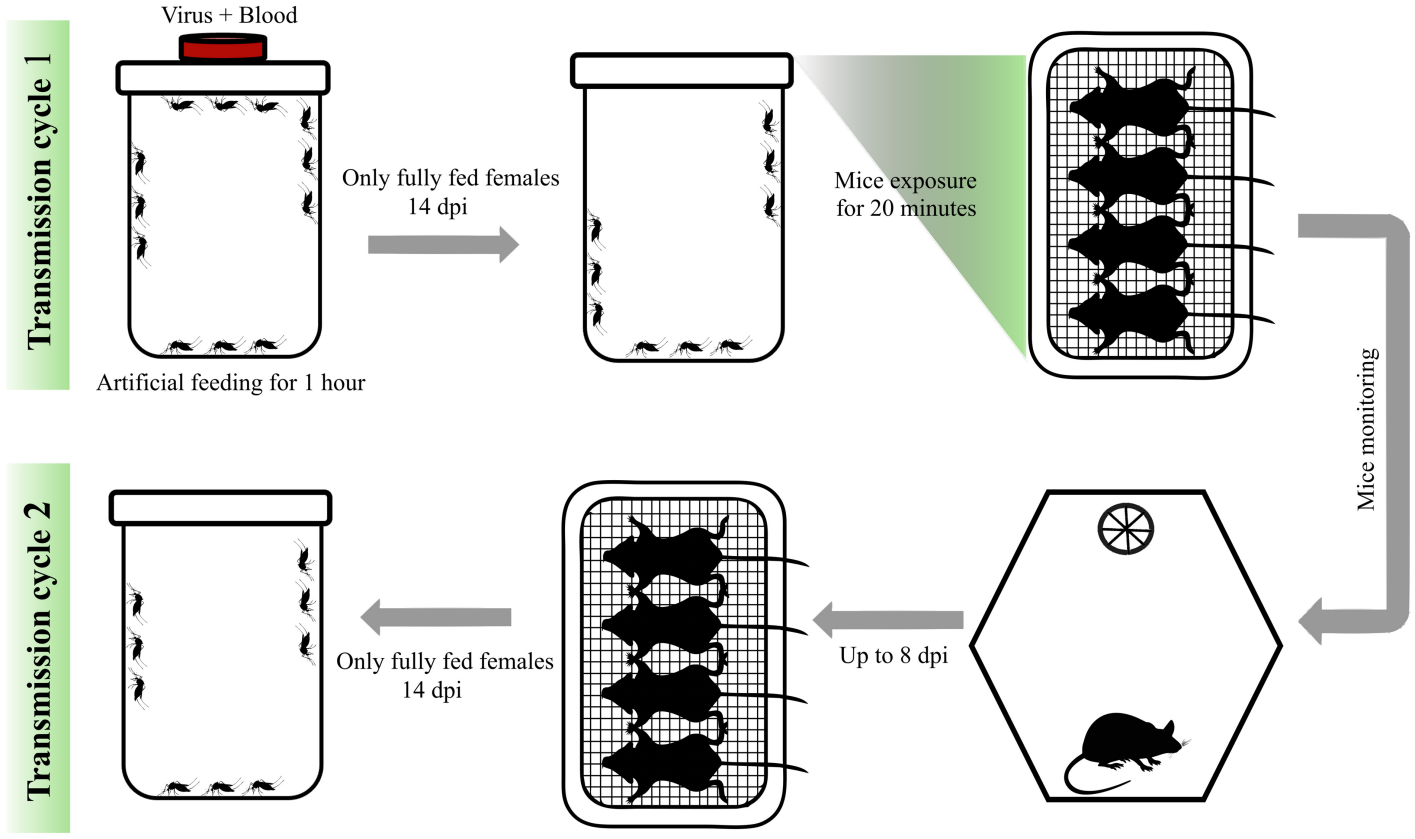
Experimental design of Zika and Zika + Mayaro transmission assay between *Aedes aegypti* and *Culex quinquefasciatus* mosquitoes and IFNAR BL/6 mice. The first stage of transmission cycle 1 involved artificially feeding mosquitoes for 60 min with a mixture of blood and virus. These mosquitoes were kept for 14 days post-infection and then exposed to groups of mice for a second blood meal, during which they transmitted the virus. The mice were anesthetized and exposed to the mosquitoes for 20 min. Animals were monitored daily for up to 8 days to assess clinical signs (viremia). Once viremia was detected, the animals were again anaesthetized and exposed to a second group of naïve mosquitoes to evaluate transmission cycle 2. These mosquitoes were also kept for another 14 days before being collected for viral detection. DPI - days post-infection.

Seven-to-ten-day-old *Ae. aegypti* and *Cx. quinquefasciatus* mosquitoes were distributed into different plastic cages comprising four groups (control and test groups for each species). The control group consisted of 100 females and 30 males, while the test group consisted of 200 females and 50 males. All experiments were conducted in three independent replicates. Both species were sugar-starved 24 h before artificial blood feeding (Supplementary Figure S1). The artificial blood-feeding assays were conducted under Biosafety Level 2 (BSL-2) conditions. Following the procedure, all plastic cages were maintained within containment units (Supplementary Figure S1). For the test group, the assays were performed using a fresh ZIKV culture harvested 72 h post-inoculation in Vero cells. For the control group, the assays were performed using uninfected Vero cell cultures. Cell culture flasks for each group were frozen at −80 °C for 20 min, then thawed at 37 °C to lyse cells and release viral particles. Cell culture was then mixed with defibrinated rabbit blood in a 1:1 ratio and ATP (Sigma-Aldrich, San Luis, Missouri, USA) in a 3 mM final concentration. The blood mixture was prepared as described by Krokovsky et al. (2023). For 60 min, the blood mixture was offered in 4 cm diameter Petri dishes containing a triple membrane of PARAFILM® M (Sigma-Aldrich, San Luis, MO, USA) on top of the cages at 37 °C using heat packs, which were changed every 20 min to maintain the temperature. Immediately after feeding, mosquitoes were cold-anesthetized, and only fully engorged females (visually sorted) were transferred to new cages to ensure feeding success (Supplementary Figure S1). The control group and test group, comprising 30 and 60 females, respectively, were named and maintained in the infection room for 14 days post-infection (dpi). At 3rd dpi, laying egg repositories were provided to the females until 7th dpi, and 24 h before the transmission assay (between mosquitoes and mice), the females were sugar-starved. Both egg laying and sugar starvation were necessary to ensure a successful second blood feeding in mice (Figure 1).

### Animal experimental design and transmission assay

The objective of transmission assays was to assess whether the viral load present in mosquitoes was sufficient to establish infection in murine tissues (transmission cycle one) and to determine whether susceptible mosquitoes could acquire infection from these animals, thereby completing the transmission cycle two.

The IFNAR/BL6 (−Ifnar1^tm1.2Ees/J^) mouse strain was obtained from the Jackson Laboratory Repository (JAX, Bar Harbor, Maine, USA) and transferred to the Central Animal Facility of the University of São Paulo, School of Medicine, Ribeirão Preto, Brazil. Animals were subsequently provided to the Animal Facility of the IAM, accompanied by the required sanitary and genetic certification. Transmission assays were conducted using IFNAR/BL6 mice aged 2–4 weeks. Both male and female mice were included. Animals were housed in micro-isolators and distributed into negative control groups (two animals) and experimental groups (four animals). Experiments were repeated three times, totalling 12 mice in the test groups and six in the control groups. All animals were maintained under controlled environmental conditions (22 ± 2 °C, 70–90% relative humidity, and a 14/12 h light/dark cycle) and acclimatized for at least 72 h before experimentation.

Fourteen days post-infection (dpi), mosquitoes were transported to the Animal Facility for challenge experiments. Prior to exposure, mice were weighed and anesthetized with a combination of ketamine (100 mg/kg; Syntec, Brazil) and xylazine (10 mg/kg; Syntec, Brazil). Anesthetized animals were then placed in a ventral position on the surface of plastic cages containing mosquitoes for 20 min to allow feeding (Figure 1; Supplementary Figure S1). During this procedure, all animals were monitored for any signs of suffering. Mosquitoes fed across the entire exposed surface of the mice, including the abdomen, chest, chin, neck, paws, and tail. All fully engorged females were collected, anesthetized at −20 °C, and individually stored in 1.5 mL RNAse/DNAse free microfuge tubes containing 300 μL of mosquito diluent (Lambrechts et al., 2009) for infection rate analysis.

After the blood feeding experiment, animals were monitored daily for up to 8 days to evaluate weight loss, clinical signs, and mortality rate (Figure 1; Supplementary Figure S1). Numerical values were assigned to the clinical signs according to the score described in Supplementary Table S1. When signs of infection were identified, the viremia period was considered as a score ≥3, mice from the test group were again anesthetized to serve as a blood source for a new group of naive mosquitoes (cycle two) with the same age and quantity as described above. After blood feeding in infected mice, mosquitoes were kept in the infection room for another 14 days and then collected as described above (Figure 1; Supplementary Figure S1). Concerning the mice group used in the second blood feeding with naïve mosquitoes, whole blood was collected, and euthanasia was carried out by cardiac puncture. After the procedure, animals were dissected to collect the brain, liver, and gonads from the negative control and test groups using sterile surgical scissors and forceps. Tissues were stored in 2 mL RNase/DNase-free tubes containing 500 μL of diluent (Lambrechts et al., 2009). Blood was centrifuged for 10 min at 2,000 g, and serum was transferred to new 1.5 mL RNAse/DNAse free microfuge tubes.

### ZIKV and MAYV coinfection and co-transmission

Infectious blood meals were offered to *Ae. aegypti* mosquitoes to investigate the dynamics of single ZIKV infection, ZIKV–MAYV coinfection, and co-transmission, using the same methodology described in both previous sections. Assays were conducted using freshly harvested ZIKV (72 h post-inoculation) and MAYV (24 h post-inoculation) cultures for the experimental group, while uninfected cultured cells were used for the control group. Virus-containing cell culture flasks were frozen at −80 °C for 20 min, rapidly thawed at 37 °C, and then mixed in equal proportions (1:1, ZIKV: MAYV). This viral mixture was subsequently combined with defibrinated rabbit blood and adenosine triphosphate (ATP; Sigma-Aldrich, St. Louis, Missouri, USA) at a final concentration of 3 mM, in a 1:1 ratio (viral inoculum: blood).

### Ethics statement

The Ethics Committee (Animal Use of the IAM) approved all animal experimental procedures with registration number CEUA/IAM 166/2021.

### Virus titration by plaque assay

Viral titer in the propagated virus (fresh) used for mosquito artificial feeding was calculated using plaque assay, as described by Krokovsky et al. (2023). The 24-well plates containing 3 × 10^5^ cells/mL in MEM supplemented with 10% FBS were prepared and incubated at 37 °C + 5% CO₂ and used 24 h after seeding. The adsorption time for ZIKV was 60 min, and for MAYV was 30 min. The plates were fixed and stained after 5 days for ZIKV and 2 days for MAYV.

#### RNA extraction and real time RT-qPCR

Mosquito and mouse tissues were individually homogenized with sterile pestles and then centrifuged for 15 min as described in Barbosa et al. (2016). RNA extraction was performed from 100 μL using TRIzol® reagent and eluted in 30 μL of nuclease-free water (Invitrogen, Carls-bad, California, USA) as described in Guedes et al. (2017). The RT-qPCR reaction was performed using the QuantiNova Kit Probe RT-PCR Kit (Qiagen, Hilden, Germany), using primers and probes, as described in Lanciotti et al. (2008) for ZIKV and Naveca et al. (2017) for MAYV. The reactions were performed in a QuantStudio 5 System (Applied BioSystems, Norwalk, Connecticut, USA) with reagents and conditions described in Krokovsky et al. (2023). All samples were evaluated for the presence of the virus in duplicate. Each RT-qPCR plate included a no-template control, a negative control from RNA extraction, and a positive control (standard curve). A standard curve used was synthesized by *in vitro* transcription using MEGAscript T7 kits (Ambion, Austin, Texas, USA) of each viral RNA and quantified in Nanodrop 2000. RNA concentration was converted into RNA copy number, as described in Kong et al. (2006). Results were analyzed using QuantStudio Design and Analysis Software v.1.3.1 with automatic threshold and baseline. Samples with Cycle quantification (Cq) values ≤ 38.5 in both duplicates were considered as positive.

#### Data analysis

The population infection rate (PIR = positive mosquitoes/total number of mosquitoes) was calculated for each species, and comparisons between PIRs were performed using Fisher’s exact test (two-tailed, 95% CI). The viral RNA copy numbers in mosquitoes were compared using the Wilcoxon test. One-way analysis of variance (ANOVA) followed by Tukey’s multiple comparison tests was used for the mouse tissue analysis in a single infection. Comparisons between single and double infection assays were performed using the Wilcoxon test. The analyzed results were considered significant when the *p*-value < 0.05. All statistical and graphical tests were performed with GraphPad Prism 8 software (GraphPad, San Diego, CA, USA).

## Results

### Single infection with ZIKV in *Ae. aegypti* and *Cx. quinquefasciatus*

**Figure 2:**
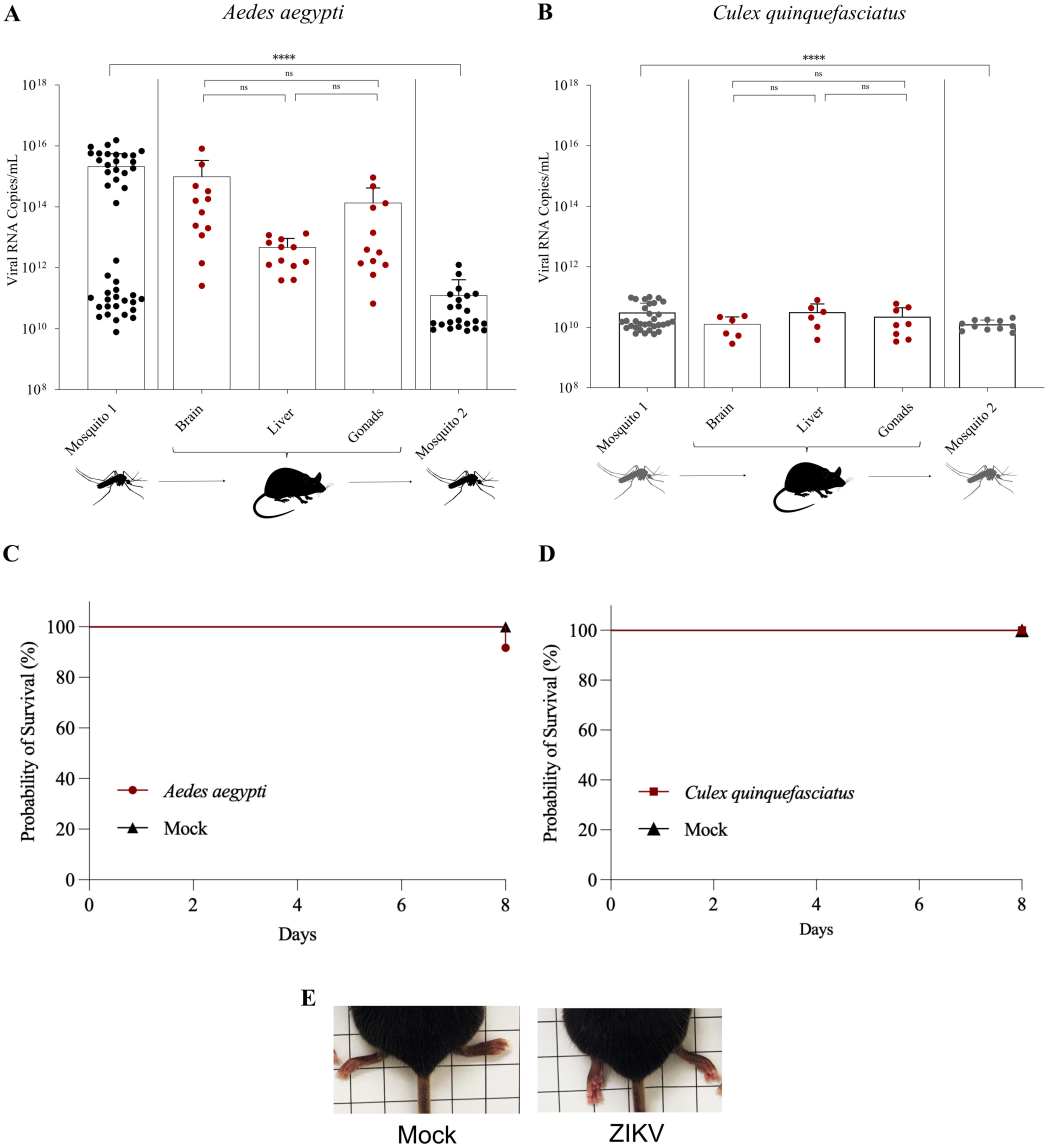
Transmission cycle panel with results of *Aedes aegypti*, *Culex quinquefasciatus,* and IFNAR BL/6 mice infected with Zika during transmission cycle one and two. **(A)** RNA copies/mL of *Ae. aegypti* (cycle one and two) and mice tissue samples infected with ZIKV. Black circles – ZIKV-positive mosquito samples; Red circles—ZIKV-positive mice samples. **(B)** RNA copies/mL of *Cx. quinquefasciatus* and mice tissue samples infected with ZIKV. Gray circles – ZIKV-positive mosquito samples; Red circles—ZIKV-positive mice samples. **(C)** Graphical representation of the probability of mice survival during ZIKV infection by *Ae. aegypti* until day eight compared with mock. **(D)** Graphical representation of the mice survival during ZIKV infection by *Cx. quinquefasciatus* until day eight compared with mock. **(E)** Paresis in the hind foot of IFNAR/BL6 mice after infection with ZIKV by *Ae. aegypti* compared to the mock. NS—not significant. Statistical analysis was performed using GraphPad Prism 8 (* *p* < 0.05, ** *p* < 0.01 and *** *p* < 0.001).

The initial titer of the mixture (ZIKV culture + blood) offered to mosquitoes ranged from 2 × 10^6^ to 5.5 × 10^6^ PFU/mL (Table 1). At 14 dpi, 45 *Ae. aeg*y*pti* and 45 *Cx. quinquefasciatus* females were allowed to blood-feed on mice. Subsequently, individual mosquitoes were collected for evaluation of populational infection rates (PIR) based on whole-body analysis (Table 1). An initial PIR of 97% (2.1 ± 3.4 × 10^15^ RNA copies/mL) was calculated for *Ae. aegypti* colony (Figure 2A) and 71% (3.08 ± 3.13 × 10^10^ RNA copies/mL) for *Cx. quinquefasciatus* (Figure 2B).

**Table 1:** Description of virus titer, populational infection rates, and transmission rates for Zika in *Aedes aegypti* and *Culex quinquefasciatus* during different experimental stages.

Species	ZIKV titer (PFU/mL)	Initial populational infection rate (mosquito-infected bloodmeal)		Transmission (infected mosquito-mice)		Final populational infection rate (infected mice-mosquito)	
		(%)	dpi	(%)	dpi	(%)	dpi
*Ae. aegypti*	3,83 ± 1,75 × 10^6^	44/45 (97.8)***†	14th	12/12 (100)	7th/8th	23/45 (51.1)*	14th
*Cx. quinquefasciatus*		32/45 (71.1)†	14th	12/12 (100)	7th/8th	11/45 (24.4)	14th

ZIKV, Zika virus; dpi, day post-infection; PFU, Plaque-forming units; mL, milliliter; %, Percentage. Virus titers are reported as mean ± standard deviation. Statistical analysis was performed using GraphPad Prism 8. Significance between mosquito species are describe as (*) *p* < 0.05 and (***) *p* < 0.001. Significance between initial and final population infection rate are discrebe as (†) *p* < 0.0001. Only significant results are shown.

A total of 36 mice were used in the experiments, including both test (*n* = 24) and control (*n* = 12) groups for both mosquito species. Among the mice (*n* = 12) exposed to *Aedes aegypti* in the test group (TG), all individuals developed clinical signs of infection at 7–8 days post-infection (dpi), including weight loss, lethargy, mortality (Figure 2C), and hind-limb paresis (Figure 2E), as detailed in Supplementary Table S2. All animals from the negative control group (NCG; *n* = 6) remained clinically healthy. In contrast, none of the mice exposed to *Cx. quinquefasciatus* from the TG (*n* = 12) or from the NCG (*n* = 6) showed signs of infection, weight loss (Figure 2D), or mortality (Figure 2F). However, despite the absence of clinical manifestations, all mice from the *Cx. quinquefasciatus* TG tested positive for ZIKV by RT-qPCR on both the 7th and 8th dpi. The description of the animals, groups, and clinical scores can be found in Supplementary Table S2.

In mice exposed to infected mosquitoes (TG), ZIKV RNA was detected in all analyzed organs, corresponding to a 100% transmission rate (Table 1). In *Aedes aegypti* experiments, the mean RNA copy number (copies/mL) was 9.9 ± 23 × 10^14^ in brain tissue, 4.7 ± 4.51 × 10^12^ in the liver, and 1.4 ± 2.8 × 10^14^ in the gonads. In *Cx. quinquefasciatus* experiments, mean RNA levels were 1.28 ± 0.9 × 10^10^ in the brain, 3.15 ± 2.73 × 10^10^ in the liver, and 2.23 ± 2.17 × 10^10^ in the gonads. Although differences in RNA copy number were observed across organs within each species, these variations were not statistically significant (Figures 2A,B). No plaque-forming units were detected in plaque assays performed on tissues from either test group.

To assess whether infected mice were capable of transmitting the virus to mosquitoes, animals previously exposed to TG mosquitoes were used as a blood source for a new cohort of naïve mosquitoes at 7- and 8-days post-infection (dpi; cycle two) for both species. These mosquitoes were collected at 14 dpi, and 45 individuals were analyzed by RT-qPCR. In this final round of analysis, 51% of *Ae. aegypti* (1.25 ± 2.77 × 10^11^ RNA copies/mL) and 24% of *Cx. quinquefasciatus* (1.25 ± 0.47 × 10^10^ RNA copies/mL) were positive for ZIKV (Table 1; Figures 2A,B).

Overall, the results from ZIKV transmission revealed a marked reduction in infection rates (IR) following viral replication in the vertebrate host. Specifically, PIR decreased by 46% in *Aedes aegypti* and by 47% in *Cx. quinquefasciatus*. Moreover, a statistically significant difference was detected in RNA copy number between samples from the primary infection (mosquitoes exposed directly to the infectious bloodmeal) and the secondary infection (mosquitoes exposed to viremic mice), as shown in Figures 2A,B.

### Coinfection and co-transmission of ZIKV and MAYV in *Ae. aegypti*

**Figure 3:**
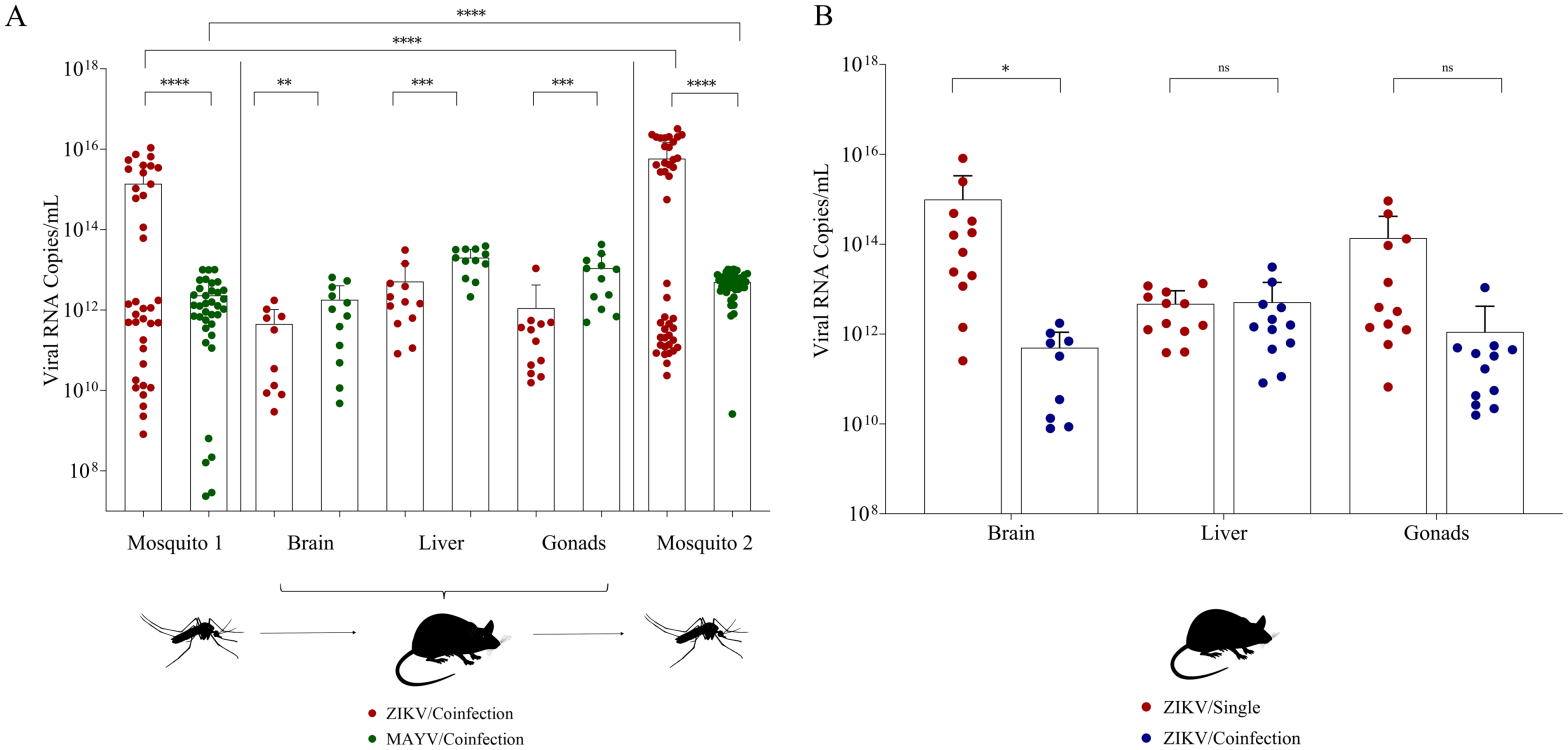
Transmission cycle panel with results of *Aedes aegypti*, *Culex quinquefasciatus* and IFNAR BL/6 mice coinfected with Zika and Mayaro. **(A)** RNA copies/mL of *Ae. aegypti* (cycles one and two) and mice tissue samples coinfected. Red circles—ZIKV positive samples; Green circles—MAYV positive samples; **(B)** RNA copies/mL of mice tissue samples infected with ZIKV and coinfected with ZIKV and MAYV. Red circles—ZIKV single infection positive samples; Blue circles—ZIKV coinfection positive samples; ns—not significant. Statistical analysis was performed using GraphPad Prism 8 (*p < 0.05, **p < 0.01 and ***p < 0.001).

**Figure 4:**
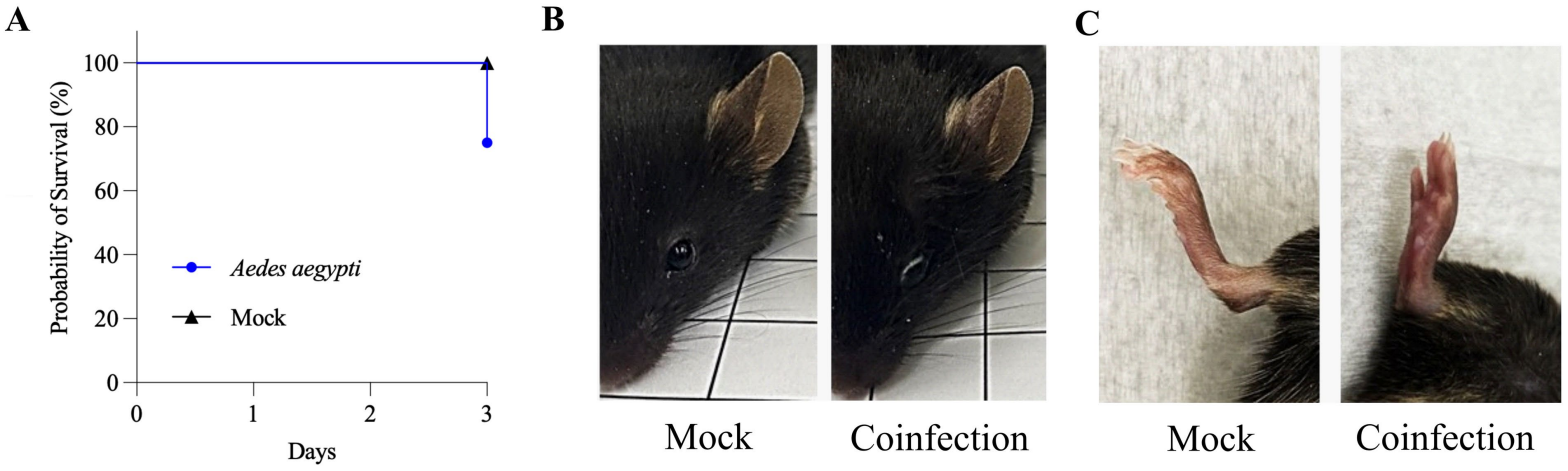
IFNAR/BL6 mice panel with results of coinfection with Zika and Mayaro by *Aedes aegypti.* **(A)** Graphical representation of the probability of survival of mice during co-infection until day three compared with mock. **(B)** Visualization of keratoconjunctivitis in IFNAR/BL6 mice after coinfection compared with mock eye. **(C)** Foot edema presented in the hind foot of mice after coinfection, compared with the mock hind foot, is shown in side view.

Regarding *Ae. aegypti* exposed to ZIKV and MAYV coinfection, the viral titer in cultures mixed with blood ranged from 2 × 10^6^ to 5.5 × 10^6^ PFU/mL for ZIKV and 2 × 10^7^ to 4 × 10^7^ PFU/mL for MAYV (Table 2). Forty-five mosquitoes were collected to calculate the PIR for both viruses. The same initial infection rate was found for both viruses (Table 2). However, the mean number of RNA copies/mL observed for these samples was 1.45 ± 2.6 × 10^15^ for ZIKV and 2.31 ± 2.85 × 10^12^ for MAYV (Figures 3A,B). During the mouse experiments, 18 animals were used on days 2 and 3. All mice from the TG (*n* = 12) showed a severe clinical evolution, with a score > 5. Animals exhibited severe weight loss, mortality (Figure 4A), and other signs such as keratoconjunctivitis (Figure 4B), and foot edema (Figure 4C). Regarding the NCG (*n* = 6), all remained healthy during the experiments. The description of the animals, groups, and clinical scores can be found in Supplementary Table S2.

**Table 2:** Description of virus titer, populational infection rates, and transmission rates for Zika and Mayaro in *Aedes aegypti* during different experimental stages.

Species	Virus	Virus titer (PFU/mL)	Initial Infection Rate (mosquito-infected bloodmeal)			Transmission (infected mosquito-mice)	Final Infection Rate (infected mice-mosquito)	
			(%)	dpi	(%)	dpi	(%)	dpi
*Ae. aegypti*	ZIKV	3,83 ± 1,75 × 10^6^	27/45 (60)	14^th^	12/12 (100)	2^nd^/3^rd^	43/45 (95.5)****	14^th^
	MAYV	3.33 ± 1.02 × 10^7^	27/45 (60)	14^th^	12/12 (100)	2^nd^/3^rd^	41/45 (91.1)**	14^th^

ZIKV, Zika virus; MAYV, Mayaro virus; dpi, day post-infection; PFU, Plaque-forming units; mL, milliliter; %, Percentage. Virus titers are reported as mean ± standard deviation. Statistical analysis was performed using GraphPad Prism 8. Significance between the initial and final population infection rate of each virus is described as (**) *p* < 0.01 and (****) *p* < 0.0001. Only significant results are shown.

Considering the animals exposed to infected *Ae. aegypti*, we observed ZIKV and MAYV replication in all analyzed organs. Regarding the number of viral RNA copies/mL, there were significantly higher values of MAYV compared to ZIKV in all organs (Figure 3A). Regarding the ZIKV replication in mice during single and coinfection, we found a higher viral replication during single infection in brain samples (Figure 3B). On days two and three of the mice infection cycle, these animals were offered as a blood source to mosquitoes, and then mosquitoes were collected at the 14th dpi. The analysis of 45 *Ae. aegypti* mosquitoes showed the successful populational infection rate for both viruses. For ZIKV, the infection rate was 95% with a mean value of 5.80 ± 8.70 × 10^15^ RNA copies/mL, while for MAYV, the infection rate was 91% with a mean value of 4.94 ± 2.71 × 10^12^ RNA copies/mL (Figures 3A,B).

The coinfection assays demonstrated elevated infection rates (IR) following viral replication in the vertebrate host. Increases of 35% for ZIKV and 31% for MAYV were observed, with statistically significant differences in RNA copy numbers between experimental groups (Table 2 and Figure 2A). Notably, analysis of the coinfection scenario indicated that MAYV led to increased ZIKV replication in both murine hosts and mosquito vectors.

## Discussion

Before the ZIKV epidemics from 2015 to 2016, only a limited number of studies had evaluated viral replication and vector competence for ZIKV across different mosquito species. An important aspect to consider is that ZIKV was first isolated from *Aedes* (Stegomyia) *africanus* (Haddow et al., 1964), and subsequent outbreaks of the virus were registered in *Ae. aegypti*-free areas (Duffy et al., 2009; Ledermann et al., 2014). This fact has initiated a series of scientific investigations into the relationship between the ZIKV and various mosquito species. Although multiple mosquito species are found infected by ZIKV in vector surveillance studies, less than 10 species can transmit ZIKV in laboratory conditions (Gutiérrez-Bugallo et al., 2019). We are aware that other factors in nature are important to allow virus transmission to sustain an outbreak, like mosquito density and anthropophily (Shaw and Catteruccia, 2019). Here, we demonstrated the ability of *Ae. aegypti* and *Cx. quinquefasciatus* to maintain a complete ZIKV transmission cycle between invertebrate and vertebrate hosts, and we also demonstrated that when a local *Ae. aegypti* colony was co-infected with ZIKV and MAYV, an increased ZIKV infection rate was observed under the co-infection condition.

The transmission model we have previously established (Krokovsky et al., 2023) was used here to understand the disparities between the vector competence of *Ae. aegypti* and *Cx. quinquefasciatus* for ZIKV and evaluate its complete transmission cycle. Regarding *Ae. aegypti*, a 97% IR was obtained after infectious bloodmeals, which led to 100% infection in mice. When naïve mosquitoes were allowed to blood feed on those animals, a 51% IR was recorded. Our data corroborate the IRs found by Guedes et al. (2017), who detected a 43% IR in *Ae. aegypti* mosquitoes at 15 dpi, and with Chouin-Carneiro et al. (2016), who evaluated diverse viral loads and temperatures, found 35 and 96% IR results for *Ae. aegypti*. Uraki et al. (2018) also investigated the transmission of ZIKV by *Ae. aegypti* in mice and obtained IR that ranged from 5 to 100% using different mosquito strains. Baldon et al. (2022) described the ZIKV transmission by *Ae. aegypti* in immunodeficient AG129 mice and found a 97% IR after a bloodmeal in viremic mice that were injected with 10^5^ PFU/mL. These data corroborate our findings, as well as when mice showed clinical signs (5–7 days) with higher severity of symptoms after 7 dpi. In addition to that, other studies used this murine model for ZIKV infection and visualized neurological signs, weight loss, and mortality between 5–8 dpi (Aliota et al., 2016; Sumathy et al., 2017).

Data on the ZIKV transmission by *Cx. quinquefasciatus* are conflicting, as vector competence studies carried out by different research groups demonstrated the refractoriness of the tested mosquito populations/colonies for the virus (Amraoui et al., 2016; Kenney et al., 2017; Lourenço-de-Oliveira et al., 2018; Main et al., 2018). On the other hand, studies in Brazil (Guedes et al., 2017), China (Guo et al., 2016), and the United States (Smartt et al., 2018) demonstrated the vector competence of *Cx. quinquefasciatus* with IR that varied between 10 and 40%, and a dissemination rate between 27 and 40%. Here, we observed an IR of 71% in *Cx. quinquefasciatus*, which resulted in successful transmission to all mice exposed (positive by RT-qPCR). Unlikely results obtained from experiments with *Ae. aegypti*, animals exposed to ZIKV-infected *Cx. quinquefasciatus* did not show any clinical signs. However, when these mice were offered as a blood source for a naïve group of mosquitoes, a 24% IR was observed. These findings reinforce the role of this mosquito as a secondary ZIKV vector and that this species can maintain the circulation of the virus in nature, even with low viral loads. This event warrants further investigation to understand why this species maintains lower viral replication. It is important to determine whether the mosquito’s immune system responds differently to ZIKV or whether the virus mutates during dissemination in *Cx. quinquefasciatus* that result in lower replication levels compared to *Ae. aegypti*. These possible interactions were observed here and in asymptomatic mice, but it is crucial to reinforce that they can serve as sources of infection for new mosquitoes of various species. Arbovirus transmission requires successful viral dissemination from the midgut to secondary tissues, particularly the salivary glands, where infectious particles can be released during blood feeding (Sanchez-Vargas et al., 2021; Blair and Olson, 2014). The transmission observed in our study demonstrates that in *Ae. aegypti*, both ZIKV and MAYV were able to overcome the midgut infection and escape barriers, disseminate through the hemocoel, and establish productive infection in the salivary glands, ultimately completing the replication cycle in a susceptible vertebrate host. In contrast, the absence of clinical signs in mice infected by *Cx. quinquefasciatus* likely reflects reduced viral dissemination efficiency within this species (Pereira et al., 2020), resulting in lower viral loads delivered during feeding. These findings reinforce the concept that vector competence is determined not only by midgut susceptibility but also by the virus’s ability to traverse dissemination barriers and achieve sufficient salivary gland titers to sustain onward transmission. The study by Guo et al. (2016) used neonatal mice to evaluate ZIKV transmission rates. These authors detected the virus in 88.9% of mice exposed to ZIKV-infected *Culex* mosquitoes. The methodological differences from each study, as well as the different viral strains and mosquito colonies, may have generated different results in the literature. This concern over vector competence studies for ZIKV has already been raised by Azar and Weaver (2019) and Wu et al., 2019, who recommended a standardization of this type of study. Here, we bring the alternative of using fresh viral stock at the time of mosquito oral infection, a viral load similar to that found in human serum, immunologically susceptible mice, and the importance of the infected mice as a source of infection for a new group of naïve mosquitoes. Although definitive standardization is difficult to apply, some viable approaches, such as those described here, can increase robustness and reproducibility (with minimum methodological criteria) in the assessment of vector competence and transmission.

Our previous study showed that *Ae. aegypti* mosquitoes were capable of transmitting MAYV to IFNAR BL/6 mice in a single infection approach (Krokovsky et al., 2023). In contrast, our previous in-depth study demonstrated that *Cx. quinquefasciatus* is unable to maintain a MAYV transmission cycle, which corroborates what has already been described in the literature (Pereira et al., 2020). In the present work, we expanded this investigation by simultaneously infecting mosquitoes with MAYV and ZIKV, thereby evaluating the complete mosquito-mice-mosquito transmission cycle. Following the infectious blood meal, mosquitoes exhibited infection rates (IR) of 60% for both MAYV and ZIKV. At 14 days post-infection (dpi), these mosquitoes successfully transmitted the viruses to 100% of IFNAR BL/6 mice. In the final stage of the cycle, naïve mosquitoes exposed to symptomatic mice reached IRs of 91% for MAYV and 95% for ZIKV. Brustolin et al. (2023) carried out a co-infection study with MAYV and ZIKV, but using *in vitro* models (Vero, Aeg2 and Huh.75 cell cultures), and using *Ae. aegypti* mosquitoes. These authors observed a decrease in ZIKV replication in the event of simultaneous infection in mosquitoes. Furthermore, previous superinfection assays conducted by the same research group demonstrated a reduction in ZIKV replication in the presence of MAYV. These findings contrast with the results observed in the present study. While the infection rate (IR) for MAYV reached 100% in single infection assays, as previously reported (Krokovsky et al., 2023) the IR observed in the current ZIKV coinfection assays was 60%, indicating a notable reduction in viral establishment under coinfection conditions. The enhanced ZIKV replication observed during co-infection with MAYV highlights that interactions between co-circulating arboviruses can modulate vector susceptibility and transmission potential, reshaping outbreak dynamics in regions where multiple viruses co-exist. These synergistic effects could accelerate viral spread, increase the number of infectious vectors, and ultimately complicate surveillance and clinical diagnosis in real-world transmission scenarios. In summary, we demonstrated that, *Ae. aegypti* is fully competent to sustain ZIKV transmission and can amplify infection under ZIKV–MAYV co-infection, whereas *Cx. quinquefasciatus* maintains only low-level ZIKV replication yet still completes the vertebrate transmission cycle.

The prevalence of *Alphavirus* replication in the presence of a *Flavivirus* has already been described in studies with mosquitoes co-infected with DENV, ZIKV, and CHIKV (Rückert et al., 2017), and in the coinfection study with ZIKV and MAYV (Brustolin et al., 2023). Our results were obtained through a blood meal from an infected host, thereby introducing additional biological variables of the virus-host-vector interaction, previously not considered, such as the immunological response. The comparison between the viral loads found in the mouse tissues derived from coinfection showed that MAYV was present in higher quantities in the liver and gonad, but no statistically significant difference was found in the brain tissue. This is supported by the well-documented ZIKV neurological tissue tropism, as described by Li et al. (2016) and Mlakar et al. (2016). When these viruses were evaluated separately, it was observed that the MAYV viral load in the liver tissue from animals submitted to ZIKV+MAYV infection was lower than that found by Krokovsky et al. (2023), who used a single infection. In the present study, there was no difference among the sampled tissues. As for ZIKV, a higher viral load was observed in brain tissue from mice infected with a single mosquito, but no difference was found in other organs. Our transmission model is designed to serve as a foundation for future experiments involving individual mice and mosquitoes, aiming to clarify and analyze infection rates, as well as behavioural studies and bite rates.

The interactions among viruses-vectors-hosts are complex and widely studied (Rückert and Ebel, 2018). In the current scenario, the co-circulation, co-infection, and co-transmission of these arboviruses have become a global health concern, since in the presence of two or more viruses, we can observe the following results: an increase or decrease in viral replication, or a null effect on replication (Vogels et al., 2019). These results reinforce that vector competence is not a binary trait but a dynamic outcome shaped by inter-viral interactions and species-specific dissemination barriers, which together have important consequences for arbovirus epidemiology in areas where multiple pathogens co-circulate. Here, we bring new insight into this interaction among viruses, mosquitoes, and a vertebrate host. After dissecting the transmission cycle of *Ae. aegypti* and *Cx. quinquefasciatus*, we were able to demonstrate the maintenance of a cycle similar to natural settings, with periods of replication and transmission lasting more than a month. A next step in this investigation would be to conduct studies like the one described here using field populations of mosquitoes and to understand viral replication in immunocompetent hosts. Finally, our findings underscore the importance of heightened vigilance regarding coinfection events, as ZIKV replication was found to increase in the presence of MAYV. Such interactions may influence viral dynamics in both vectors and hosts, potentially complicating diagnosis, clinical outcomes, and surveillance efforts. These results are particularly relevant in the current context of arbovirus co-circulation in Brazil and other South American countries, where overlapping transmission cycles may pose additional challenges for public health strategies.

## Data Availability

The original contributions presented in the study are included in the article/Supplementary material, further inquiries can be directed to the corresponding author.
