# Supplementary material for: Dissecting Zika virus transmission by Aedes aegypti and Culex quinquefasciatus mosquitoes to a vertebrate host and its coinfection with Mayaro virus
Source: Front Microbiol. 2026 Jan 28;16:1724153. doi: 10.3389/fmicb.2025.1724153 (PMC12891122; doi:10.3389/fmicb.2025.1724153)
Supplement: Supplementary file 1 [file Supplementary_file_1.docx]

**Supplementary Information**

**Dissecting Zika virus transmission by *Aedes aegypti* and *Culex quinquefasciatus* mosquitoes to a vertebrate host and its coinfection with Mayaro**


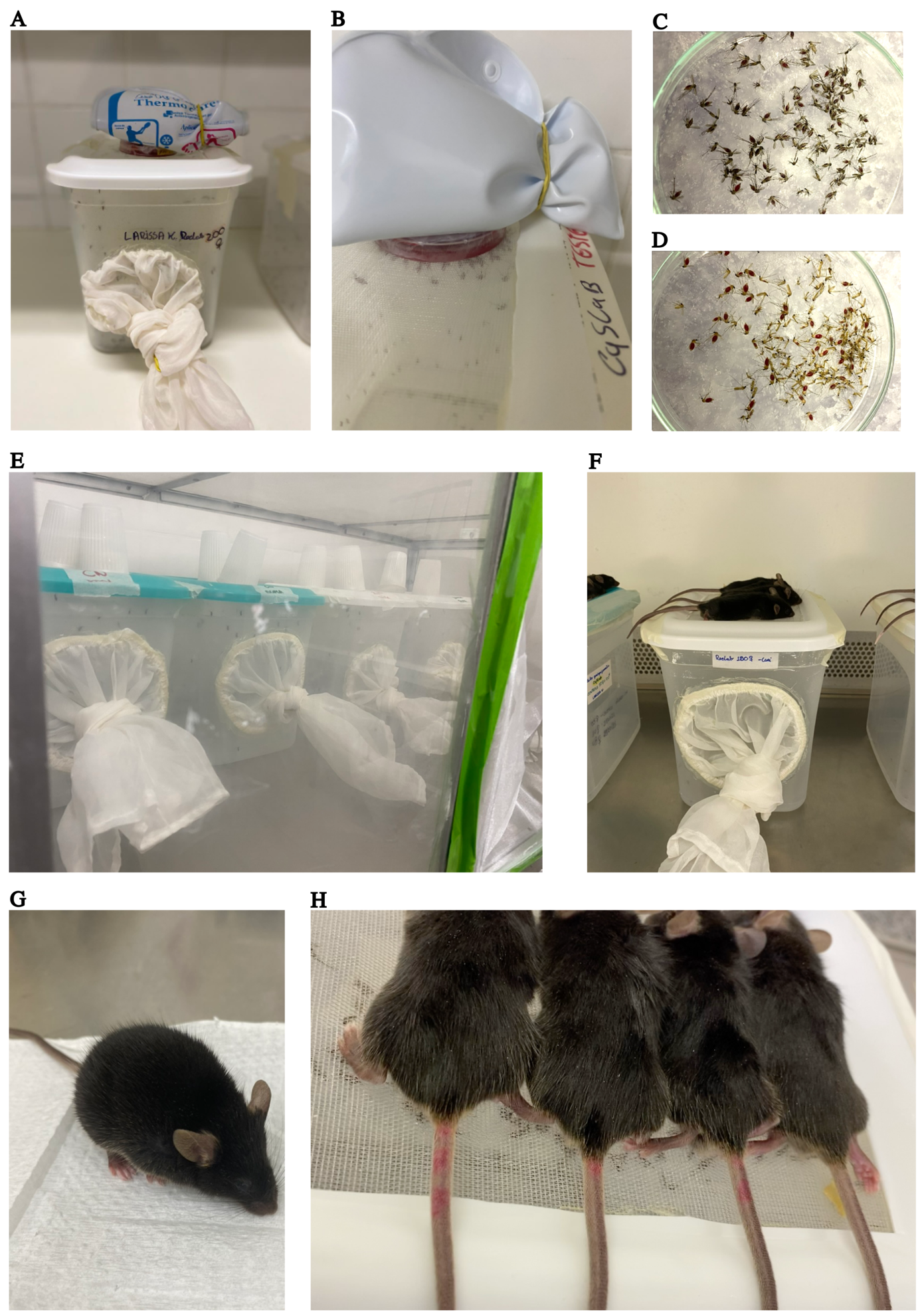
**Supplementary figure 1:** Panel of photographs captured during transmission experiments of Zika and Zika+Mayaro viruses between *Aedes aegypti* and *Culex quinquefasciatus* mosquitoes, and IFNAR/Bl6 mice. (A) Artificial mosquito feeding system consisting of a plastic cage with mosquitoes, a petri dish with a blood+virus mixture and a thermal pack. (B) Close-up photograph of the artificial mosquito feeding system showing engorged mosquitoes. (C) Anesthetized *Aedes aegypti* mosquitoes on ice for the separation of the fully engorged females. (D) Anesthetized *Culex quinquefasciatus* mosquitoes on ice for the separation of the fully engorged females. (E) Plastic cages inside the containment cage in the Biosafety Level 2 Laboratory. (F) Mice exposure to mosquitoes previously artificially fed inside a Biosafety Cabinet (BSC) in the Biosafety Level 2 Laboratory in the Animal Facility. (G) Mice during evaluation for signs of disease, with ruffled hair, lethargy and edematous hind paw. (H) Close-up photograph to visualize the mosquitoes feeding from the naive group (cycle 2) on mice after assessment of signs of disease.

**Supplementary Table 1:** Table with a list of clinical signs of infection and related *score* for IFNAR/Bl6 mice evaluation.

| **Clinical Signs** | ***Score*** |
| --- | --- |
| Isolation | 1 |
| Ruffled hair | 1 |
| Mild lethargy | 1 |
| Dehydration | 1 |
| Curved posture | 1 |
| Moderate lethargy | 2 |
| Weight loss (≥10%) | 2 |
| Weight loss (≥20%) | 3 |
| Foot edema | 3 |
| Keratoconjunctivitis | 3 |
| Paresis and Paralysis | 3 |
| Severe lethargy | 3 |
| Diarrhea | 3 |

**Supplementary Table 2:** Description of the experimental groups and details of the weights, clinical signs and score of the IFNAR/Bl6 mice during transmission assays.

| **Virus/replicate** | **Mice Sex** | **Mice ID** | **Initial weight (g)** | **Final weight (g)** | **Dpi** | **Clinical Signs** | **Score** |
| --- | --- | --- | --- | --- | --- | --- | --- |
| ***Aedes aegypti*** | | | | | | | |
| ZIKV/01 | Male | NCG01 | 18.9 | 18.8 | 7^th^ | No | 0 |
|  |  | NCG02 | 17.8 | 18.3 | 7^th^ | No | 0 |
|  |  | TG01 | 19.5 | 18.3 | 7^th^ | Isolation, curved posture, moderate lethargy, weight loss (≥10%) | 7 |
|  |  | TG02 | 21.3 | 19.1 | 7^th^ | Isolation, wight loss (≥10%) | 3 |
|  |  | TG03 | 17.6 | 13.5 | 7^th^ | Isolation, curved posture, moderate lethargy, weight loss (≥10%) | 7 |
|  |  | TG04 | 20.4 | 18.3 | 7^th^ | Isolation, wight loss (≥10%) | 3 |
| ZIKV/02 | Female | NCG01 | 16.8 | 17.2 | 7^th^ | No | 0 |
|  |  | NCG02 | 15.7 | 16.1 | 7^th^ | No | 0 |
|  |  | TG01 | 17.8 | 15.4 | 7^th^ | Isolation, weight loss (≥10%) | 3 |
|  |  | TG02 | 16.6 | 13.8 | 7^th^ | Isolation, weight loss (≥10%) | 3 |
|  |  | TG03 | 17.6 | 16.6 | 7^th^ | Isolation, weight loss (≥10%) | 3 |
|  |  | TG04 | 17.2 | 17.1 | 7^th^ | Isolation, weight loss (≥10%) | 3 |
| ZIKV/03 | Female | NCG01 | 17.6 | 18.5 | 8^th^ | No | 0 |
|  |  | NCG02 | 19.7 | 20.2 | 8^th^ | No | 0 |
|  |  | TG01 | 20.9 | 16.1 | 8^th^ | Isolation, mild lethargy, weight loss (≥10%), paresis nerve paresis | 7 |
|  |  | TG02 | 22.6 | 21.6 | 8^th^ | Isolation, mild lethargy, wight loss (≥10%) | 4 |
|  |  | TG03 | 22.1 | 17.9 | 8^th^ | Isolation, mild lethargy, wight loss (≥10%) | 4 |
|  |  | TG04 | 19.1 | 22.9 | 8^th^ | Isolation, mild lethargy, wight loss (≥10%) | 4 |
| ***Culex quinquefasciatus*** | | | | | | | |
| ZIKV/01 | Female | NCG01 | 14.5 | 15.1 | 7^th^ | No | 0 |
|  |  | NCG02 | 14.5 | 14.3 | 7^th^ | No | 0 |
|  |  | TG01 | 15.1 | 15.7 | 7^th^ | No | 0 |
|  |  | TG02 | 16.2 | 16.1 | 7^th^ | No | 0 |
|  |  | TG03 | 14.9 | 15.6 | 7^th^ | No | 0 |
|  |  | TG04 | 16.2 | 16.8 | 7^th^ | No | 0 |
| ZIKV/02 | Male | NCG01 | 20.3 | 21.1 | 7^th^ | No | 0 |
|  |  | NCG02 | 18 | 19.5 | 7^th^ | No | 0 |
|  |  | TG01 | 17.9 | 20.6 | 7^th^ | No | 0 |
|  |  | TG02 | 17.1 | 19.5 | 7^th^ | No | 0 |
|  |  | TG03 | 16.7 | 22.1 | 7^th^ | No | 0 |
|  |  | TG04 | 16.8 | 23 | 7^th^ | No | 0 |
| ZIKV/03 | Male | NCG01 | 21.4 | 21.3 | 8^th^ | No | 0 |
|  |  | NCG02 | 22.4 | 22.8 | 8^th^ | No | 0 |
|  |  | TG01 | 21.5 | 21.9 | 8^th^ | No | 0 |
|  |  | TG02 | 22.1 | 22.5 | 8^th^ | No | 0 |
|  |  | TG03 | 23.4 | 23.7 | 8^th^ | No | 0 |
|  |  | TG04 | 20.3 | 20.5 | 8^th^ | No | 0 |
| ***Aedes aegypti*** | | | | | | | |
| ZIKV+MAYV/01 | Male | NCG01 | 22.1 | 22.1 | 3^rd^ | No | 0 |
|  |  | NCG02 | 21.1 | 21.4 | 3^rd^ | No | 0 |
|  |  | TG01 | 20.9 | 19.2 | 3^rd^ | Isolation, curved posture, dehydration, weight loss (>10%), moderate lethargy | 7 |
|  |  | TG02 | 21.9 | - | 3^rd^ | Death | - |
|  |  | TG03 | 21.4 | 18.9 | 3^rd^ | Isolation, curved posture, dehydration, weight loss (>10%), moderate lethargy | 7 |
|  |  | TG04 | 20.4 | - | 3^rd^ | Death | - |
| ZIKV+MAYV/02 | Female | NCG01 | 16.9 | 16.7 | 2^nd^ | No | 0 |
|  |  | NCG02 | 15.2 | 15.4 | 2^nd^ | No | 0 |
|  |  | TG01 | 20.2 | 17.2 | 2^nd^ | Ruffled hair, curved posture, dehydration, weight loss (>10%), moderate lethargy | 7 |
|  |  | TG02 | 18.7 | 16.7 | 2^nd^ | Ruffled hair, curved posture, dehydration, weight loss (>10%), moderate lethargy | 7 |
|  |  | TG03 | 22.1 | 16.2 | 2^nd^ | Ruffled hair, curved posture, dehydration, weight loss (>10%), moderate lethargy | 7 |
|  |  | TG04 | 22.8 | 15.7 | 2^nd^ | Ruffled hair, curved posture, dehydration, weight loss (>10%), moderate lethargy | 7 |
| ZIKV+MAYV/03 | Female | NCG01 | 17.6 | 17.2 | 2^nd^ | No | 0 |
|  |  | NCG02 | 17.1 | 16.9 | 2^nd^ | No | 0 |
|  |  | TG01 | 17.3 | 15.4 | 2^nd^ | Ruffled hair, curved posture, dehydration, weight loss (>10%), moderate lethargy | 7 |
|  |  | TG02 | 16.9 | 15.3 | 2^nd^ | Ruffled hair, curved posture, dehydration, weight loss (>10%), moderate lethargy | 7 |
|  |  | TG03 | 17.2 | 15.6 | 2^nd^ | Ruffled hair, curved posture, dehydration, weight loss (>10%), moderate lethargy, keratoconjunctivitis | 10 |
|  |  | TG04 | 19.0 | 17.4 | 2^nd^ | Ruffled hair, curved posture, dehydration, weight loss (>10%), moderate lethargy, keratoconjunctivitis | 10 |
